# Trends in the use of coercive measures in Finnish psychiatric hospitals: a register analysis of the past two decades

**DOI:** 10.1186/s12888-019-2200-x

**Published:** 2019-07-26

**Authors:** Maritta Välimäki, Min Yang, Tero Vahlberg, Tella Lantta, Virve Pekurinen, Minna Anttila, Sharon-Lise Normand

**Affiliations:** 1 Department of Nursing Science, Faculty of Medicine, 20014 University of Turku, Turku, Finland; 2 School of Nursing, Hong Kong Polytechnic University, Hong Kong, Hong Kong, Special Administrative Region People’s Republic of China; 3 Turku University Hospital, Turku, Finland; 4 West China Research Center for Rural Health Development, Sichuan University Huaxi Medical Center, Sichuan University of China, Administration Building, No 17, Section 3, Ren Ming Nan Lu, Chengdu, Sichuan China; 5 Department of Biostatistics, University of Turku, 20014 University of Turku, Turku, Finland; 6 Department of Health Care Policy, Harvard Medical School, Boston, USA; 7 Department of Biostatistics, Harvard T.H. Chan School of Public Health, Boston, USA

**Keywords:** Psychiatry, Aggression, Trends, Coercive measures, Register

## Abstract

**Background:**

Coercive measures is a topic that has long been discussed in the field of psychiatry. Despite global reports of reductions in the use of restraint episodes due to new regulations, it is still questionable if practices have really changed over time. For this study, we examined the rates of coercive measures in the inpatient population of psychiatric care providers across Finland to identify changing trends as well as variations in such trends by region.

**Methods:**

In this nationwide registry analysis, we extracted patient data from the national database (The Finnish National Care Register for Health Care) over a 20-year period. We included adult patients admitted to psychiatric units (care providers) and focused on patients who had faced coercive measures (seclusion, limb restraints, forced injection and physical restraints) during their hospital stay. Multilevel logistical models (a polynomial model of quadratic form) were used to examine trends in prevalence of any coercive measures as well as the other four specified coercive measures over time, and to investigate variation in such trends among care providers and regions.

**Results:**

Between 1995 and 2014, the dataset contained 226,948 inpatients who had been admitted during the 20-year time frame (505,169 treatment periods). The overall prevalence of coercive treatment on inpatients was 9.8%, with a small decrease during 2011–2014. The overall prevalence of seclusion, limb restraints, forced injection and physical restraints on inpatients was 6.9, 3.8, 2.6 and 0.8%, respectively. Only the use of limb restraints showed a downward trend over time. Geographic and care provider variations in specific coercive measures used were also observed.

**Conclusions:**

Despite the decreasing national level of coercive measures used in Finnish psychiatric hospitals, the overall reduction has been small during the last two decades. These results have implications on the future development of structured guidelines and interventions for preventing and more effectively managing challenging situations. Clinical guidelines and staff education related to the use of coercive measures should be critically assessed to ensure that the staff members working with vulnerable patient populations in psychiatric hospitals are ethically competent.

**Electronic supplementary material:**

The online version of this article (10.1186/s12888-019-2200-x) contains supplementary material, which is available to authorized users.

## Background

The deinstitutionalisation of psychiatric services has led to an increase in international discussion [1–3] about human rights and coercive practices in psychiatric hospitals. The degree of deinstitutionalisation in psychiatric services in Finland has been among the highest across Europe [4]. As a result of this, the number of psychiatric beds decreased by about 50% between the years 1993 and 2011 [5]. Rapid changes in the treatment system have meant new opportunities for reformed out-patient care and patient social participation [6]. Currently, the number of out-patient treatment periods in psychiatric specialised care is almost 12 times the number of treatment periods in hospital care (2.25 million treatment periods versus 195 000 periods, respectively) [7].

Despite of structural and ideological changes in mental health service, seclusion, restraint, and forced medication is being used in many psychiatric hospitals [8–11]. However, the recent results in reducing the use of coercive measures are promising [12–15]. Less restrictive interventions have been used to prevent and manage patient violent behaviour, including de-escalation techniques [16], improving collaboration between patients and nurses, developing organisational culture and safe ward environments [17–20] and training staff members [21]. Although patient-related factors, like psychotic symptoms [22], organic mental disorder [23], young age [24] and perceived coercion [22] have been found to increase the risk to be coerced during hospital admission, the factors associated with patient coercion are more complex. For example, the staff’s priority in treatment may emphasise safety issues and would therefore have a higher acceptance for intrusive measures than patients [25, 26]. Other factors related to the treatment environment were found in a study by Pettit et al. [27]. Based on their findings, the authors concluded that the availability of a seclusion room may be related to the use of seclusion, as staff members are more likely to consider seclusion as an acceptable method of managing aggressive incidents when there is a designated space for it.

On the other hand, understanding the realities of patient coercion is challenging due to differing registration systems, daily practices [28, 29] and staff attitudes towards the use of coercion [30, 31]. Therefore, decreasing the use of coercive measures in daily practice may be challenging. In Finland, the Ministry of Social Affairs and Health launched the National Mental Health Policy in 2009 to reduce the need for using coercive measures in psychiatric services [32]. After strong initiatives and the implementation of the national strategy between 2006 and 2013, substantial decreases in the use of seclusion rooms (incidence rate ~ 30%, a prevalence of 40 to 27/100,000 inhabitants) and mechanical restraints (incidence rate ~ 38%, a prevalence of 24 to 14/100,000 inhabitants) were reported [33], although there is still room for improvement, considering the corresponding numbers in other Scandinavian countries. On the other hand, in the broader international context, the use of coercive measures in Finland is at a satisfactory level [28, 34].

Despite a reduction in the prevalence of coercion practices in Finland, there is still a need to better understand the current trends of the use of patient coercive measures due to ethical and practical issues [35]. Further, a systematic review has concluded [36] that more research is needed to formally evaluate the outcomes of the implementation of risk monitoring systems and to assess their effectiveness in health services regarding use of coercive measures. Therefore, in this study we ask three specific research questions. First, are the change trends for the different coercive measures similar? Second, is the use of coercive measures the same in different regions? Third, if the use of coercive measures is not the same in the different regions, how does it vary? The answers to these questions may fill the gap in our understanding of the context and lead to the future improvement of service at the national level. To our knowledge, this is the first nationwide study based on non-selected register data on actual use of coercive measures and its variation over two decades. We focus on inpatient psychiatric care because, in these services, the use of coercive measures is allowed under the Mental Health Act, 1116/1990 [37]. We examined trends between 1995 and 2014 because, for that time period, the reporting system of patient coercive practices is more consistent between hospitals and years.

In this study and for the psychiatric patient population, we first examined the overall prevalence rates of coercive use, both generally and by specific type of coercive measures. Based on the strong emphasis in Finland to decrease the use of coercive measures in psychiatric hospitals [32], we assumed that we would find evidence that the prevalence of coercive measures used in psychiatric hospitals had decreased during the last two decades. We further identified the trends in prevalence rates of coercive measures use on all inpatients admitted during 1995 to 2014, stratified by gender. Finally, we used a multilevel modelling approach, which took into account that patients were nested within care providers, and that patient age was confounded with the treatment period. We investigated the trends in the change of prevalence rates, looking at the difference between gender and geographic areas, and variations across psychiatric care providers.

## Methods

### Study population and settings

The study population was confined to all adult patients admitted to psychiatric hospitals. In 2014, the number of psychiatric beds was 19.9 in psychiatric hospitals and 48.6 in psychiatric units in general hospitals per 100,000 inhabitants [38]. As a Nordic country with 5.5 million inhabitants, the service structure for mental health care is challenging because the country is geographically large and sparsely populated, where a third of the population lives in the capital city area [39]. Social welfare and health care services are implemented by municipalities with government support, who are responsible for the prevention, early diagnosis, treatment, and rehabilitation of mental health disorders [40]. The financing of these services is mostly tax-based; the costs are paid by the state, the municipalities, the Social Insurance Institution Kela and by private parties, such as households and insurance companies [41]. The private sector provides services particularly in the area of psychotherapy. In addition, mental health services are provided by NGOs [42].

The data of this current study is based on the registers of health care providers under the Ministry of Social Affairs and Health where coercive measures are permitted based on the Finnish Mental Health Act, 1116/1990 [37]. These include psychiatric wards, clinics and rehabilitation services. In these units, the Ministry of Social Affairs and Health is responsible for the regulations for admission to treatment and other official governance of mental health services. We included all adult patients (18 years old and older) who were admitted into psychiatric inpatient services in Finland between 1 January 1995 to 31 December 2014 and whose medical information had been registered in the Finnish National Care Register for Health Care, HILMO [43]. The study population included patients who had a Finnish Personal Identification Code, namely Finnish citizens and foreigners (with a residence permit valid for at least 1 year) who were living in Finland permanently, and also foreigners without a Finnish Personal Identification Code who had been treated in these psychiatric hospitals during the study period.

Units offering only forensic psychiatric care were excluded from the study. Treatment in these units differs significantly from that of general psychiatric services, e.g., in terms of length of treatment periods; whereas in general psychiatric inpatient care, the average duration of a treatment period has been 36 days [42], in forensic psychiatric care, the average treatment duration is over 4 years [44]. In Finland, a total of 7 units provide forensic mental treatment and carry out mental examinations on adults [45]. In 2013, a total of 498 patients were admitted for mental examination or compulsory forensic mental health hospital care [44]. In early 2019, the total bed capacity of forensic units was around 600. Regarding two major forensic inpatient care providers, i.e., the two state mental health hospitals, there have not been any significant changes in the number of forensic beds in the past 20 years [46–48].

We also excluded psychogeriatric units because current psychogeriatric treatment is mostly provided in long-term non-psychiatric care institutions, which are not regulated under mental health services [49]. Also currently, after the era of deinstitutionalisation in Finland, only a few hospital districts offer inpatient psychiatric services specialised in psychogeriatric treatment [50], but the exact statistics of these hospital beds is not available. Further, the use of coercive measures in non-psychiatric units is not regulated under the Mental Health Act [51], and therefore, exact statistics regarding coercive measures in those units is not available.

### Data sources

The Finnish National Care Register for Health Care, HILMO [43] (originally, the National Hospital Discharge Register, NHDR, 1969–1993), maintained by the Finnish Institute of Health and Welfare, was our primary data source. The data for each patient can be identified based on a unique personal identification code [43]. The Care Register identifies information about care providers (hospital/clinic), the route of admission to psychiatric inpatient care, the number of hospital days, the length of involuntary care, the number of admissions to psychiatric inpatient care, global functioning GAS [52] at admission and discharge, medication, and coercive measures used [43]. According to a literature review by Sund [53], the completeness and accuracy of the register were found to vary from ‘satisfactory’ to ‘very good’. Most of the studies included examined validity regarding diagnoses of, for example, vascular disease, injuries and mental disorders. The studies performed validation by checking that certain disease cases could be identified from the register. The proportion of register-detected diagnoses that were confirmed to be true-positives by the study specific external data varied between 75 and 99% for common diagnoses. However, regarding rare diseases, the false positives were found to be more likely, which was thought to be related to the uncertainty in clinical diagnoses. Nevertheless, clear recording errors were found to be quite rare [53].

### Patient, regional and care provider-level variables

What constitutes coercive measures was defined and identified based on the Finnish Mental Health Act, 1116/1990 [37]. These measures can only be used during a patient’s involuntary treatment or while a patient has been admitted to hospital for observation. Treatment against a patient’s will may last for a maximum of 3 months (may be continued with a new treatment order, if needed). *Seclusion* refers to the decision to isolate a patient from other patients, which can be made by an attending physician on the basis of an examination of the patient performed by a physician. In urgent cases, other members of the health care staff may, on a temporary basis, seclude a patient, after which the matter must immediately be communicated to the physician. *Limb restraint* is when a patient may be tied down with belts or comparable tools if other measures are not sufficient. *Forced injection* may be used only if the failure to do so would seriously jeopardise the health and safety of the patient or others. The attending physician decides on the medication that is involuntarily given. However, the Finnish Mental Health Act [37] does not regulate which classes of medication are allowed to be used as forced medication. On the other hand, the Finnish Regional State Administrative Agencies have stated that the use of depot injections is prohibited when a patient has been admitted to hospital for observation, to determine whether or not the conditions for ordering a person to undergo treatment against their will are met [54]. In psychiatric clinical practice, intramuscular medications often include both first (e.g. haloperidol) and second (e.g. olanzapine) generation antipsychotics, and benzodiazepines (mainly lorazepam) [55]. *Physical restraint* involves holding the patient as is necessary to seclude the patient for therapeutic reasons.

Individual patients with a primary psychiatric diagnosis based on an ICD-9 [56] or ICD-10 [57] classification, who were admitted into an adult psychiatric treatment unit, were identified in the national health register by the information specialist (at the National Institute of Health and Welfare) using unique personal identification code. The data of each individual patient were collected, including information on gender, age, admission and discharge dates, and coercive measures used (seclusion, limb restraint, forced injection and physical restraint [yes, no]) [43]. Patient information was managed in two categories: information about individual patients and information about treatment periods. The latter option was needed in the analysis because a patient may have been admitted to inpatient psychiatric care more than one time; this can be identified in the data when the number of treatment periods in a specific year is higher than the number of patients admitted into the hospital. After data identification from the national register, each patient identification code was encrypted.

Information about specific care providers (which includes multiple wards on the hospital or clinic level) was first collected from The Care Register for Health Care for specialised psychiatric care based on unique care provider codes regarding each patient admission. The specific location of each care provider by municipality was then identified from the public care provider register (TOPI), maintained by the National Institute of Health and Welfare [58], based on the unique care provider codes. The municipalities (location) of each care provider were then further classified into five regions according to the NUTS 2008 regional classification system (Nomenclature of Territorial Units for Statistics; [59]) by using a converter tool provided by Statistics of Finland [60]. The NUTS regional classification, which consists of NUTS levels 1–3, was formulated by the European Union, and is followed in the compilation of all common regional statistics of the EU [61]. We used the NUTS 2 classification into five regions, because its purpose is to utilise the applications of regional policies on a national level [62]. In 2014, the population rates of each region were: Southern Finland (2 765 094 inhabitants), Western Finland (1 377 281), Eastern Finland (641 346), Northern Finland (659 116) and Åland (28 916) (31.12.2014 [63]). For possible effects of the treatment environment, we created a psychiatric care provider level variable: the number of treatment periods in different years at each care provider.

### Statistical methods

#### The study design and sample size

We calculated the prevalence of coercive measures based on patient units identified by unit codes in the register data. To present the time trend, we considered the admission year to be the time frame, assuming that the groups of patients in each time frame consisted of a cross-sectional sample population. Regarding admissions that crossed the new year, we included the coercive measures and patients in the statistics describing the year the admission started, because we had no information about the specific time when coercive measures had been used. One patient could have been admitted repeatedly to different care providers in more than one region at different times. This design considers one patient who might have had multiple admissions to the same care provider in 1 year as one unit, but if the patient was submitted to two or more care providers during one calendar year, he or she is counted as two or more units. Likewise, one patient would be counted as two or more units if he or she was admitted to the care provider or different care providers in two or more calendar years. This design gave us a concise data structure where patient units were nested within psychiatric care providers, allowing one patient to represent repeated units over 20 calendar years, allowing us to estimate the variation in the prevalence of coercive measures toward patients among care providers by region, and making it possible to observe changing trends over time.

Based on a unique inpatient identification code, the dataset contained 226,948 inpatients over the 20-year period. Among those inpatients, 46,539 (20.5%) were admitted to two or more hospitals in at least 1 year and had multiple treatment periods. These patient units totalled 293,497 in the sample. Among these patient units, 51,312 (17.5%) were admitted in two calendar years, 21,079 (7.2%) were admitted in three calendar years, and 8.2% were admitted in four to 20 calendar years. Assuming independent patient samples from different care providers in different time frames, the complete cross-sectional sample for the final analysis was 505,169 patient units or observations.

#### Dependent variable

The main dependent variable was the use of any coercive measure on a patient unit, which was defined in binary form and coded as 1 if the patient unit had received any coercive measure, and as 0 otherwise. Because each patient unit could potentially face multiple episodes of different coercive measures, we also defined the four specific measures (seclusion, limb restraints, forced injection and physical restraint) separately in binary form. These coercive measures were not mutually exclusive (more than one type of method could be used in one treatment period), hence each measure was analysed as a separate dependent variable.

#### Models

To examine trends in prevalence of any coercive measure as well as the four specific coercive measures over time, and to investigate variation in such trends among care providers and regions, multilevel logistical models for the analysis were used.

Let *j* indicate hospitals (at level 2), and *i* indicate patient units (at level 1). Then, the log-odds of dependent variable *y*_ij_ as a function of time/year with random effects of intercepts (u_0j_) and time effects (u_1j_ and u_2j_) among care providers can be expressed as follows:

$$ {\displaystyle \begin{array}{l}{y}_{ij}={\beta}_{0j}\left(\operatorname{int} ercept\right)+{\beta}_{1j}{(year)}_{ij}+{\beta}_{2j}{\left( year\hat{\mkern6mu} 2\right)}_{ij}\\ {}{\beta}_{0j}={\beta}_0+{u}_{0j},\kern0.5em {\beta}_{1j}={\beta}_1+{u}_{1j},\kern0.5em {\beta}_{2j}={\beta}_2+{u}_{2j}\\ {}\left[\begin{array}{c}{u}_{0j}\\ {}{u}_{1j}\\ {}{u}_{2j}\end{array}\right]\sim \kern0.5em MN\left(0,V\right),\kern0.5em V=\left[\begin{array}{ccc}{\sigma}_{u_0}^2& & \\ {}{\sigma}_{u_{01}}& {\sigma}_{u_1}^2& \\ {}{\sigma}_{u_{02}}& {\sigma}_{u_{12}}& {\sigma}_{u_2}^2\end{array}\right]\end{array}} $$

A polynomial model of the quadratic form was used to describe an overall time trend of dependent variables in the model. The year variable was recoded as a continuous scale ranging from 1 to 20, to correspond to each year from 1995 to 2014. The model allowed random effects u_0j_, u_1j_ and u_2j_ of mean estimates for the intercept, and the linear slope and quadratic slope to vary across hospitals respectively. The random effects (u_0j,_ u_1j_ and u_2j_) were assumed from multivariate normal distribution with a variance-covariance matrix V in which there were six random coefficients for their variances and covariance to be estimated. These coefficients allowed us to test how much the time trend potentially varied among hospitals. We used a generalised Ward test for this purpose. A penalised quasi-linearisation procedure was used for the estimation [64]. Binomial variance of the dependent variable was checked by estimating and testing the model dispersion parameter at level 1. Normality of random effects was checked using Q-Q plots for their marginal distribution.

To examine gender differences in the time trends, we tested gender as a covariate and interaction terms between gender and the slopes β_1_ and β_2_. In the same way, time trend differences among regions were fitted and tested in the model. Age and treatment periods of psychiatric care providers were treated as confounding factors and adjusted in the modelling analysis.

Descriptive analyses were conducted using SAS System for Windows version 9.4 [65] and multilevel models were applied using MLwiN v2.35 [66].

## Results

### Description of the data

Between 1 January 1995 and 31 December 2014, there were 505,169 patient observations from 92 different psychiatric care providers (different care provider codes) in the data. Patients were treated mostly (49.5%) at care providers located in Southern Finland, and only 0.5% in the Åland region. The number of patients declined over time, with an increase in the proportion of female patients admitted, as shown in Table 1. The age of patients was about 44 years old during the 20-year period. The overall prevalence of any coercive measures used on inpatients was 9.8% with small decrease during 2010–2014. The overall prevalence of seclusion, limb restraints, forced injection and physical restraints on inpatients was 6.9, 3.8, 2.6 and 0.8%, respectively. Only the use of limb restraints showed a trend of declining over time, starting in 2000–2004, and other measure presented small nonlinear changes in their overall prevalence. Among patients who received coercive measures (*N* = 49731), 69% experienced one type of measure, and 21.9, 7.2 and 1.9% experienced two, three and four measures, respectively. There was little change over time in the frequency of using multiple measures on patients.

**Table 1:** Descriptive statistics of study population by region by time period

	Admission time period				
	1995–1999	2000–2004	2005–2009	2010–2014	Total
Patients by regions: N(%)					
Southern Finland	64479(50.1)	72788(52.2)	60835(47.2)	51965(48.0)	250067(49.5)
Western Finland	27810(21.6)	29085(20.9)	30701(23.9)	26365(24.3)	113961(22.6)
Eastern Finland	19713(15.3)	20036(14.4)	19482(15.1)	14527(13.4)	73758(14.6)
Northern Finland	16270(12.6)	16919(12.2)	16987(13.2)	14774(13.6)	64950(12.9)
Åland	497(0.4)	475(0.3)	747(0.6)	714(0.7)	2433(0.5)
All regions	128769(100.0)	139303(100.0)	128752(100.0)	108345(100.0)	505169(100.0)
Female patient: %	45.8	47.7	49.2	50.0	48.1
Patient age: mean ± SD	43.4 ± 15.3	43.3 ± 15.5	44.2 ± 16.3	43.9 ± 17.2	43.7 ± 16.0
Patients received coercive measures by type: N(%)					
Seclusion	8129(6.3)	9489(6.8)	9896(7.7)	7206(6.7)	34720(6.9)
Limb restraints	5209(4.1)	5891(4.2)	4851(3.8)	3162(2.9)	19113(3.8)
Forced injection	3160(2.4)	3763(2.7)	3056(2.4)	2913(2.7)	12892(2.6)
Physical restraints	1295(1.0)	1071(0.8)	696(0.5)	882(0.8)	3944(0.8)
Any coercive method	12598(9.8)	13919(10.0)	13240(10.3)	9974(9.2)	49731(9.8)
Patients received multiple coercive measures: N(%)					
None	116171(90.2)	125384(90.0)	115512(89.7)	98371(90.8)	455438(90.2)
One method	8915(6.9)	9312(6.7)	9218(7.2)	6850(6.3)	34295(6.8)
Two methods	2478(1.9)	3227(2.3)	2974(2.3)	2199(2.0)	10878(2.2)
Three methods	898(0.7)	1072(0.8)	859(0.7)	785(0.7)	3614(0.8)
Four methods	307(0.2)	308(0.2)	189(0.2)	140(0.1)	944(0.2)
Patients received any coercive measures by region: N(%)					
Southern Finland	5629(8.7)	7674(10.5)	6678(11.00)	5056(9.7)	250067(10.0)
Western Finland	2955(10.6)	2736(9.4)	3151(10.3)	2472(9.4)	113961(9.9)
Eastern Finland	2607(13.2)	2262(11.3)	2223(11.4)	1360(9.4)	73758(11.5)
Northern Finland	1390(8.5)	1231 (7.3)	1142(6.7)	1029(7.0)	64950(7.4)
Åland	17(3.4)	16(3.4)	46(6.16)	57(8.0)	2433(5.6)

We observed much variation among regions in Finland regarding the overall prevalence of using coercive measures. The overall prevalence was similar between Southern and Western Finland, with 10% (95% CI, 9.9–10.1%) vs. 9.9% (95% CI, 9.7–10.1%). The highest prevalence was in Eastern Finland (95% CI, 11.3–11.7%), and the lowest was in the Åland region (95% CI, 4.7–6.5%). The next lowest prevalence was in Northern Finland (95% CI, 7.2–7.6%).

### Gender differences in coercive measures

The overall prevalence of any coercive measures for male patients was 11.23%, and the prevalence of seclusion, limb restraints, forced injection and physical restraints for males was 8.02, 4.75, 2.41 and 0.78% respectively. For female patients, the overall prevalence was 8.35%, and for the specific measures, prevalence was 5.63, 2.74, 2.71 and 0.79%, respectively (see Additional file 1: Table S2). The model adjusted the odds-ratio of female patients based on the estimation in Table 2: 0.70 (95% CI 0.68–0.72), 0.67 (0.65–0.70), 0.54 (0.52–0.57), 1.09 (1.04–1.16) and 1.01 (0.92–1.12), respectively, for overall prevalence and then the four specific measures. This confirmed a significantly lower overall prevalence of seclusion and limb restraints having been used on female patients compared to that of males, a higher prevalence of forced injection, and no gender difference in physical restraints.

**Table 2:** Gender difference in model estimates of time trends in log-odds by coercive measures (standard error in brackets)

	Any coercive measure	Seclusion	Limb restraints	Forced injection	Physical restraints
Reference group					
Intercept	−5.43(.795)‡	−5.92(.907) ‡	−6.49(1.04)‡	−7.62(.919)‡	−5.55(.757)‡
Year	−.0014(.0136)	.012(.014)	−.014(.020)	.016(.016)	−.043(.022)*
Year^2	−.0057(.0019)†	−.0072(.0020)†	−.014(.0006)‡	−.0057(.0023)*	−.0089(.0026)†
Female vs Reference					
Intercept	−.357(.014)‡	−.396(.017)‡	−.608(.022)‡	.093(.027)‡	.012(.050)
Year	−.013(.002)‡	−.016(.002)‡	−.013(.003)‡	−.017(.003)‡	.0063(.0055)
Year^2	.0008(.0003)†	.0009(.0004)†	.0005(.0006)	−.0007(.0062)	−.0008(.0011)

Estimates were adjusted for age, gender and treatment periods of care providers using a multilevel logistic model with random intercepts and random slopes of linear and quadratic terms. The reference group consists of male patients of a median age of 40 from hospitals with minimum treatment periods in Southern Finland. The significant result was based on a Z-score test for each parameter estimate: ‡*p* < 0.001, †*p* < 0.01, *p < 0.05

**Fig. 1:**
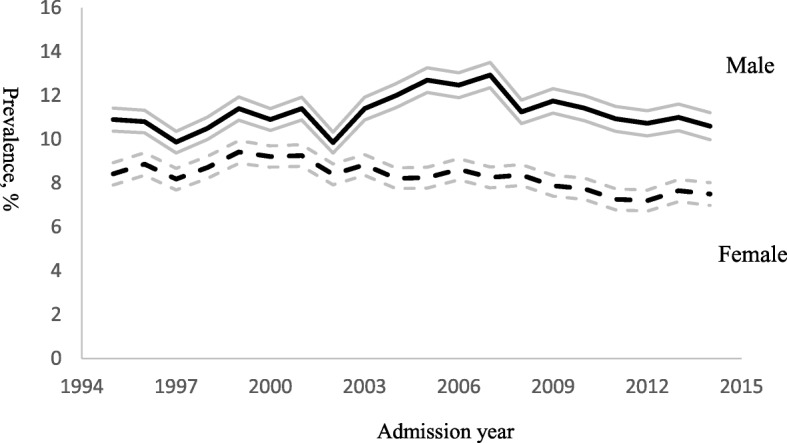
Time trend of prevalence of any coercive treatment method by patient gender with 95% confidence intervals (CI): raw data

The time trends in Fig. 1 show a consistent declining trend among female patients with both model-estimated linear and quadratic change rates being significantly different from male patients (see Table 2). By 2014, the gap in prevalence between genders was greater than it was 20 years prior.

**Fig. 2:**
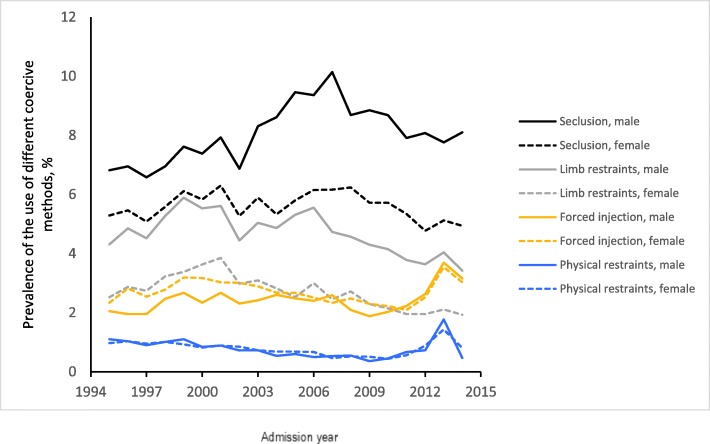
Gender difference in time trend of prevalence of different coercive treatment methods: raw data

The raw time trends of the four specific coercive measures by gender are shown in Fig. 2 and model-estimated gender differences in time trends are presented in Table 2. The model estimates suggest a significant difference in the linear and quadratic terms between genders in the use of seclusion, limb restraints and forced injection, but no statistical gender difference in the use of physical restraints over time. Both raw data and model analysis showed a consistent pattern of the difference in time trends between genders.

### Change trends for different coercive measures

**Fig. 3:**
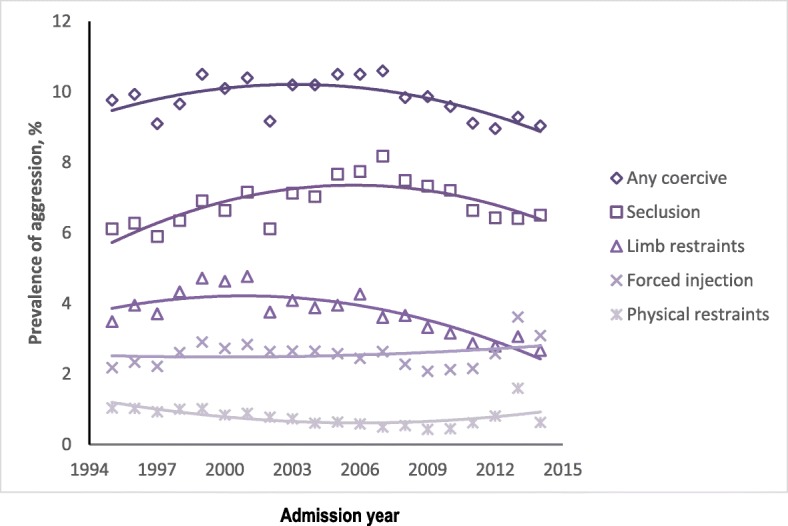
Time trend of prevalence of different coercive treatment methods: raw data and smoothed curve by quadratic function

Combining both genders, Fig. 3 presents the raw prevalence of coercive measures used over time with the yearly prevalence shown in Additional file 1: Table S1. For any coercive measure, the trend was fractural from 1995 to 1999, flattened at around 10% during 2000–2007, and then gradually decreased from 2008 to 2014 at around 9%. The non-linear declining trend is seen with the fitted quadratic model, with the linear slope estimated as 0.18% per year (SE = 0.057, *p* = 0.0015), and the accelerated declining slope as − 0.011% (SE = 0.0029, *p* = 0.0002). Because 49.2% of coercive measures were categorised as seclusion, the trend of change in the rate of seclusion is similar to that of the overall rate. The rate of limb restraints shows a consistent decline from its peak at 4.77% in 2001 to 2.67% in 2014, with the linear slope estimated as 0.121 (SE = 0.045, *p* = 0.007) and the non-linear slope as − 0.010 (SE = 0.002, p = 0.000). On the other hand, the use of forced injection had a rather flat trend in time, with no significant linear and quadratic slope estimates (*p* = 0.79 and 0.55 respectively), but an increased rate from 2010 to 2013. Finally, physical restraints rose from 2010 to 2013 but then experienced a downturn in 2014 with a significant change in slopes for both linear and quadratic terms over time (*p* = 0.0007 and 0.003 respectively).

**Fig. 4:**
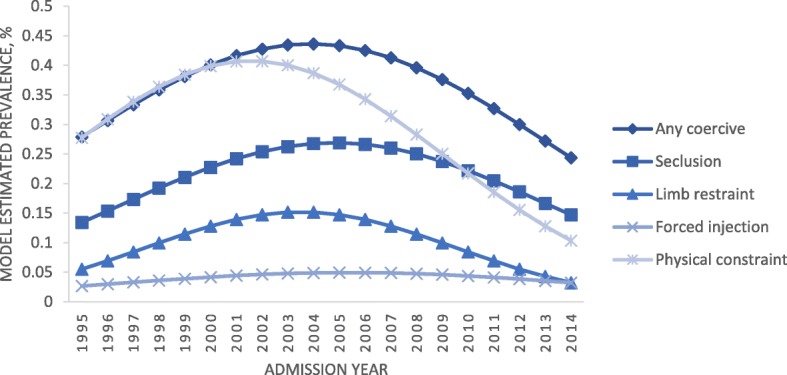
Model-estimated prevalence trends by type of coercive treatment methods (based on the reference group of patients: male in 40 years from hospitals with minimum treatment periods in Southern Finland region)

The model-estimated change trend of each measure presented in Fig. 4 further confirms the quadratic change of time trends for any coercive measure, seclusion and limb restraints, but shows a different trend from the raw rate for forced injection and physical restraints, after controlling for effects of age, gender, number of treatment periods of care providers and regions.

### Regional differences

A total of 92 care providers distributed over five geographic regions of the country were analysed in this study: Southern Finland, Western Finland, Eastern Finland, Northern Finland and the Åland islands. The distribution of inpatients in the five regions was 49.5, 22.6, 14.6, 12.9 and 0.5%, and the raw rate of any coercive use in the 20-year period was 10.0, 9.9, 11.5, 7.4 and 5.6%, in the five regions respectively. Regarding the time trends, the rate of any coercive use presents a ‘∩’ shape for Southern Finland and a ‘∪’ shape for Northern Finland, a slow decline from 11.2 to 9.1% in Western Finland, a considerable decline from 14.1 to 9.2% in Eastern Finland, and a fractural increase in Åland (see Additional file 1: Table S3).

After adjusting for age, gender and treatment periods of care providers, and taking into account random intercepts and slopes among care providers, the comparison between Southern Finland and the other four regions in the change patterns of the coercive measures by model estimates in Table 3 suggest the following findings. Firstly, there was no significant difference in the change pattern of any coercive measure rate (estimates in the second column of Table 3) among regions. For both seclusion and forced injection, a significantly faster change in the quadratic nonlinear rate was found in Northern Finland than in Southern Finland (Odds 1.002 vs 0.99, *p* = 0.037 for seclusion; Odds 1.004 vs 0.994, *p* = 0.026 for forced injection). This statistical difference may not have clinical importance. Other regions did not show statistical differences in the trends of the two measures. For limb restraint measures, Western, Eastern and Northern Finland presented an increased quadratic nonlinear change rate, compared to Southern Finland. The estimated odds for Southern Finland were 0.986, compared with that of Western Finland at 0.996 (*p* < 0.0001), of Eastern Finland at 1 (*p* < 0.0001), and of Northern Finland at 1.001 (*p* < 0.0001). Finally, Western and Northern Finland also showed some differences from the southern region in the same term regarding the use of physical restraints. The Åland region did differ from Southern Finland in any of the five measures, which could be due to a small number of inpatients.

**Table 3:** Regional difference in model estimates of time trends in log-odds by coercive measure (standard error in brackets)

	Any coercive measure	Seclusion	Limb restraints	Forced injection	Physical restraints
Reference group					
Intercept	−5.43(.795)‡	−5.92(.907) ‡	−6.49(1.04)‡	−7.62(.919)‡	−5.55(.757)‡
Year	−.0014(.0136)	.012(.014)	−.014(.020)	.016(.016)	−.043(.022)*
Year^2	−.0057(.0019)†	−.0072(.0020)†	−.014(.0006)‡	−.0057(.0023)*	−.0089(.0026)†
Western Finland vs Reference					
Intercept	−.500(.323)	−.588(.367)	−.693(.400)	−.458(.366)	−.149(.318)†
Year	.0006(.026)	.0021(.026)	−.015(.040)	−.0062(.028)	.039(.037)
Year^2	.0045(.0037)	.0065(.0040)	.010(.0010)*	.0077(.0043)	.018(.0044)†
Eastern Finland vs Reference					
Intercept	.276(.339)	.263(.384)	.915(.415)	.071(.378)	.363(.337)
Year	.0031(.028)	−.0066(.029)	−.0086(.039)	−.025(.031)	−.039(.041)
Year^2	.0025(.0038)	.000032(.0041)	.014(.0009)‡	.0054(.0045)	.0031(.0048)
Northern Finland vs Reference					
Intercept	−.898(.374)	−1.85(.433)	−.436(.462)	−1.46(.428)†	−.639(.374)
Year	.0085(.030)	.0309(.032)	.029(.044)	−.044(.034)	−.032(.042)
Year^2	.0068(.0041)	.0092(.0046)*	.015(.001)‡	.010(.0045)*	.014(.0051)*
Åland vs Reference					
Intercept	−.293(.920)	−.306(1.07)	−1.03(1.20)	.255(1.07)	.317(.996)
Year	.079(.078)	.081(.080)	.220(.131)	.111(.084)	−.111(.097)
Year^2	.011(.011)	.011(.011)	.015(.012)	.012(.012)	.017(.012)

Estimates were adjusted for age, gender and treatment periods of care providers using a multilevel logistic regression model with random intercepts and random slopes of linear and quadratic terms. The reference group consisted of male patients of a median age of 40 from hospitals with minimum treatment periods in Southern Finland. The significant result was based on a Z-score test for each parameter estimate: ‡*p* < 0.001, †*p* < 0.01, *p < 0.05

### Variation among care providers

Ninety-two care providers (based on the care provider code) were distributed in the five regions by a percentage of 46.7% (*n* = 43), 21.7% (*n* = 20), 16.3% (*n* = 15), 13.0% (*n* = 12) and 2.2% (*n* = 2), respectively. The final model estimates in Table 4 indicate that the time trends of all coercive measures vary significantly among care providers. For example, the odds of variation of interception of any coercive measure among care providers was 3.13 (95% CI: 2.17–4.50), the odds of variation of linear slope of the year was 1.006 (95% CI: 1.004–1.008), and that of the quadratic change slope of the year was 1.0001 (95% CI: 1.00008–1.00016). For limb restraints, only the random intercepts and random linear slopes were found to significantly vary among care providers.

**Table 4:** Estimates of random coefficients in terms of variance and covariance of time trend parameters among care providers

Random coefficients	Any coercive measure Est (SE)	Seclusion Est (SE)	Limb restraints Est (SE)	Forced injection Est (SE)	Physical restraints Est (SE)
Var: Intercepts	1.14(.186)**‡**	1.45(.238)**‡**	1.68(.281)**‡**	1.31(.227) **‡**	0.931(.176)**‡**
Cov:Year/Intercept	−.018(.011)	−.032(.012)	.0087(.019)	−.0071(.013)	−.014(.015)
Var: Year	.0057(.0011)**‡**	.0056(.0012)*	.129(.025)**‡**	.0058(.0013)**†**	.010(.002)**‡**
Cov: Year^2/Intercept	.0018(.0015)	.024(.0018)		−.0055(.0020)*	.0063(.0019)**†**
Cov: Year^2/Year	−.0006(.0001)**‡**	−.0004(.0001)*		−.0004(.00015)**†**	.00017(.00019)
Var: Year^2	.00012(.00002)**‡**	.00013(.00003)**‡**		.00013(.00003)**‡**	.00011(.00003)**†**
χ^2^ statistic (*p* value)	51.00 (0.000)	47.86 (0.000)	27.10 (0.000)	40.33 (0.000)	33.68 (0.000)

The model with random slopes of quadratic terms did not converge for limb restraints; hence, only a random linear slope model was fitted on this method. The χ^2^ statistic was based on a generalised Ward test for joint parameter estimates using the MLwiN package. ‡*p* < 0.001, †*p* < 0.01, *p < 0.05

**Fig. 5:**
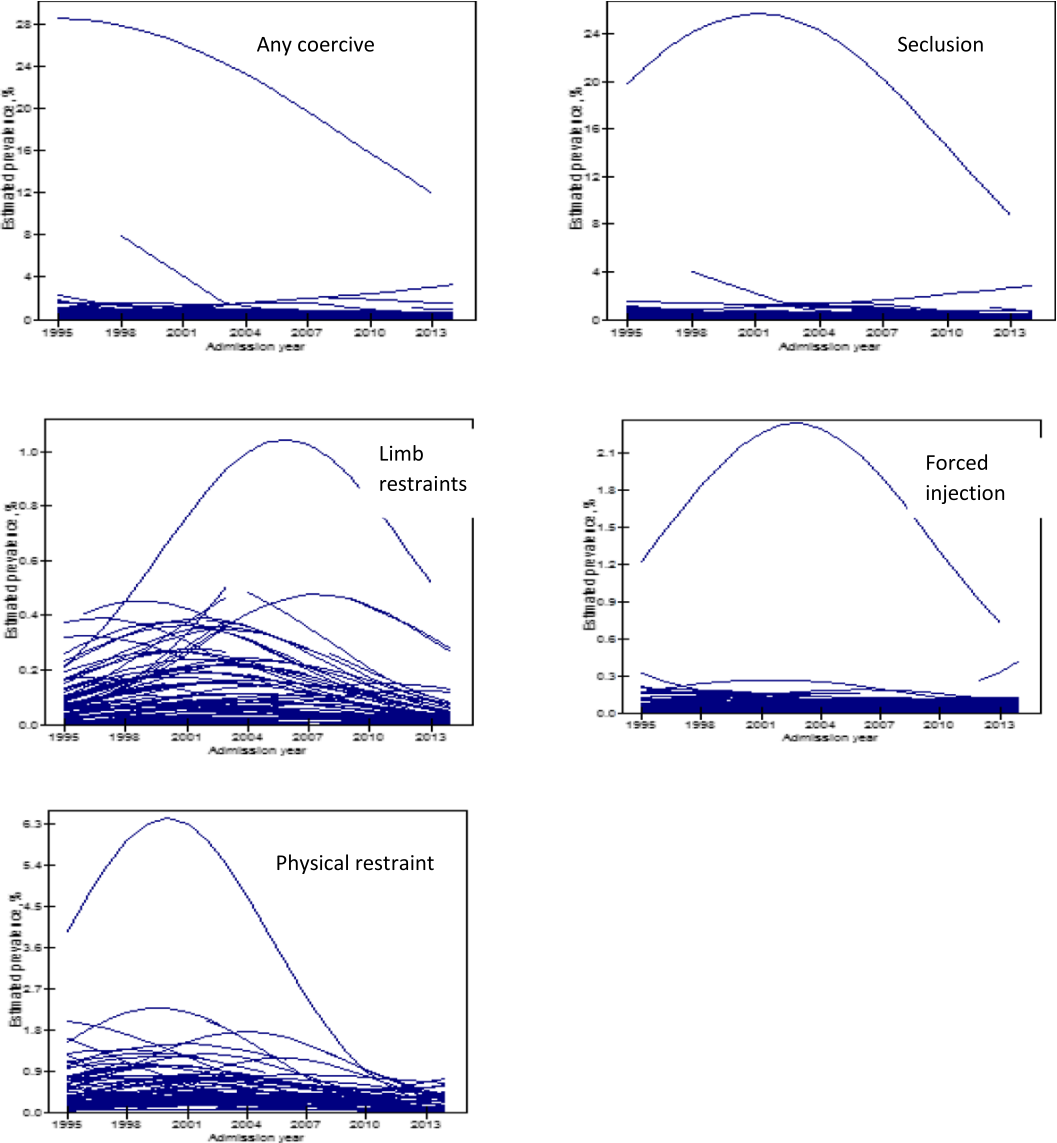
Model-estimated prevalence (%) of coercive treatment methods by care providers (each line represents a predicted time trend of coercive treatment use in each care provider or hospital, based on the fully adjusted model)

Further visualising the model predicted time trends of each outcome measures by care providers (Fig. 5), we found that the estimated variation was mainly due to one particular care provider, where a small number of patients were treated. If this care provider is removed, the variation in the time trends of coercive measures among the rest of the care providers is small or moderate.

## Discussion

Our study provides novel insight into how coercive measures have been used during the last two decades in Finnish psychiatric treatment services. This is the first study where coercive measures have been analysed, in detail and over such a long period of time, based on national register data. We assumed that we would find evidence that the prevalence of coercive measures used in psychiatric hospitals has decreased during the last two decades. Despite a decline in the number of patients admitted into psychiatric hospitals, the number of coerced patients has not decreased in the same manner. This finding is somewhat contradictory to our preliminary assumption.

In our study, we did not observe a linear declining trend in the decrease of coercive measures. At the same time, the data showed that the trends in the use of different coercive measures are changing. Seclusion is still the most commonly used coercive measure in psychiatric hospitals, and the rate of limb restraints shows a consistent decline (1.86-fold reduction from 5.10 in 2001 to 2.74% in 2014). However, the use of forced injection and physical restraints rose from 2010 to 2013, but went down in 2014. There are a few assumptions which may shed some light on our findings. First, compared to many other countries, the proportion of psychiatrists and nursing staff in Finnish mental health services per 100,000 inhabitants is one of the highest in the world [67]. Therefore, the high number of patient coercion cannot be explained by the lack of manpower. We can ask, though, whether staff have enough knowledge to use the alternative methods for managing patient aggression [68]. On the other hand, the statistics show that in Finland approximately €150 million (including indirect costs) has already been invested in continuing education in health and social services for staff every year [69], although in 2015, the average expenditure by enterprises on continuing vocational training courses in 28 EU countries was higher than that in Finland (1,418 purchasing power standards per participant vs. 1,257 in Finland) [70]. Second, staff may have favourable attitudes toward intrusive measures because of a strong emphasis on safety issues at work [25, 26]. However, in Finland, the decision of whether the need for treatment, or the harmfulness of the patient’s behaviour is serious enough to justify coercion to ensure safety is made based on clinical judgement [71]. Therefore, a more systematic analysis of the use of coercive measures should be conducted on a national level. The development of the practices should also follow the principles set by the United Nations’ Convention on the Rights of Persons with Disabilities (CRPD) [72]. In addition, national clinical guidelines could harmonise practices by giving recommendations based on existing evidence at the highest level gathered from interventions that have already been carried out [14, 19, 21].

Our model-estimated and adjusted trend shows an increased prevalence rate from 1995 to a peak in 2001, and a slow decline from 2002 to 2007, followed by a consistent linear decrease from 2007 to 2014. Radical changes in coercion rates from 2001 to 2002 may be the result of new regulations in the Mental Health Act, 1423/2001 [73], specifically regarding the use of seclusion and restraint. An explicit regulation about the use of mechanical restraint and seclusion has been included in the Mental Health Act since 2002 [28], which aims to define specific reasons for limiting the rights of involuntarily treated patients as well as to standardise coercive measures nationwide [8]. Indications of using coercive measures may explain the sharp downturn in the rates of these measures, and the continuing declining trend in the rate of coercive measures after 2002. In 2008, the European Committee for the Prevention of Torture and Inhuman or Degrading Treatment or Punishment (CPT) once again visited Finland. As an outcome of the visit, the committee required Finnish authorities to urgently provide a detailed action plan to significantly reduce the frequency and duration of using patient seclusion [74]; this could be another explanation for the downturn. Further, the national action plan for 2009–2015 [75] aimed to increase awareness of the importance of reducing coercive measures by organising national meetings and workshops and increasing the awareness of staff about the need to decrease coercive measures. Still, the reduction in the overall use of coercive measures is quite small. From 1995 to 2014, there was a 1.11-fold reduction in the rate from 10.2 to 9.2/100 inpatients. Although national and regional guidelines and acts have offered a direction for the changes in clinical practices during the last two decades, these approaches may not be integrated into current practices.

We found in our study a significantly lower overall prevalence of seclusion and limb restraints use on female patients compared to that of males. On the contrary, female patients had a higher prevalence of forced injection. Georgieva et al. [76] conducted a randomised clinical trial to evaluate whether seclusion and coercive incidents would be reduced by using involuntary medication. The authors conclude, based on their findings, that although the use of involuntary medication could successfully replace and reduce the number of seclusions, alternative interventions are needed to reduce the overall number and duration of coercive incidents [76]. Therefore, keeping this finding in mind, a wider variation of treatment alternatives should be found to prevent and manage challenging situations on the wards. These alternatives could include non-invasive methods, such as the use of a ‘soft room’ or a one-on-one patient sitter, which may be more easily accepted by patients [77]. Using alternative methods would be important because patients themselves have found the use of coercive measures to be frustrating, traumatising [78], distressing [79], and a less-than-humane experience [80]. Having these negative experiences may increase the risk of non-adherence in treatment, especially for young males [81]. On the contrary, if staff members perceive coercive measures as curative, not too much personal tension by staff members has been put on the current situation. We can also ask why changes in patient coercion practices have not happened in Finland corresponding with economic growth in the country [82], higher education levels [83] and the high level of well-being of residents [84]. Finland also has the highest human capital in the world [85], and the quality of health care and provisions are very good [86]. Further, Finland has been named as the world’s safest country [87], where people are highly satisfied with life [88].

If a failure to markedly reduce the use of coercive measures is explained by the lack of awareness of the value of treatment culture or by the identification of those patients who are specifically at risk to be coerced, we urgently need to be aware of factors related to each patient’s risk to be coerced in order to develop interventions for reducing the use of coercion in the future [22]. The importance of conducting meetings with psychiatrists, nurses and patients after the use of coercive measures has also been highlighted [89]. Further, family members should also be included in the treatment system when aiming to reduce involuntary treatment [90]. While several studies have shown that it is possible to develop treatment systems using novel interventions [14, 19, 21], more emphasis should be put on evidence-based interventions to reduce coercion in psychiatry.

### Strengths and limitations

Using data from a nationwide register gives the unique opportunity to get a deeper understanding of general trends in Finnish society. Our sample included all adult patients who were hospitalised as inpatients in a psychiatric care providers in Finland during a 20-year period. Methodological biases should be considered. First, the definitions [3, 91] and legislations [92] used to describe and guide patient coercive measures in psychiatric settings may vary, which may restrict the generalisability of the study results for various geographical areas such as in Åland, possibly due to the small number of patients each year. Second, our register data were retrospectively collected, and therefore the data may provide more a follow-up type of information, which always includes a risk for biases, such as mistakes in data collection [93].

## Conclusions

Although national policies and legislative changes in Finland may have had positive affects by decreasing the use of coercive measures, clear, lasting changes in patient coercive measures have not yet been achieved. Our study demonstrates the importance of developing clinical practices toward interventions designed to reduce patient aggressive events and the use of coercive measures. Therefore, in the future, there should be a shift on ongoing clinical monitoring, using more humane interventions. In this effort, information technology could have an important role [94]. A critical analysis of daily practices in psychiatric hospital care in line with specific coercive measures used is needed, and should be supported by more targeted and effective on-the-job education about human interaction and safety issues. Further research is also needed on identifying factors that might be associated with an increase in coercive measures at the regional, hospital and patient levels, combined with widening the understanding of daily practices in psychiatric care. This could increase transparency and reduce the closed institutional culture [95].

## Additional file

Additional file 1: **Table S1.** Number of inpatients and prevalence of coercive measures among inpatients by admission year: raw data. **Table S2.** Prevalence of coercive measures among inpatients by gender by admission year: raw data. **Table S3.** Number of inpatients and prevalence of any coercive measures among inpatients by admission year and region. **Table S4.** Estimates of all parameters by multilevel logistic regression models with random effects (SE in brackets). (DOCX 40 kb)

## Data Availability

The data that support the findings of this study are available from The Finnish National Care Register for Health Care but restrictions apply to the availability of these data. The data is usable for this study only. Professor Maritta Välimäki confirms that she had full access to all the data in the study, and takes responsibility for the integrity of the data and the accuracy of the data analysis.

## Abbreviations

CPT: Committee for the Prevention of Torture
CRPD: Convention on the Rights of Persons with Disabilities
GAS: Global Assessment Scale
HILMO: The Finnish National Care Register for Health Care
ICD-10: International Statistical Classification of Diseases and Related Health Problems 10th Revision
ICD-9: Classification of Diseases and Injuries
ID: Identification Code
NHDR: The National Hospital Discharge Register
NUTS: Nomenclature of Territorial Units for Statistics
