# Supplementary material for: Trends in the use of coercive measures in Finnish psychiatric hospitals: a register analysis of the past two decades
Source: BMC Psychiatry. 2019 Jul 26;19:230. doi: 10.1186/s12888-019-2200-x (PMC6660969; doi:10.1186/s12888-019-2200-x)
Supplement: Supplementary file 1 — Table S1. Number of inpatients and prevalence of coercive measures among inpatients by admission year: raw data. Table S2. Prevalence of coercive measures among inpatients by gender by admission year: raw data. Table S3. Number of inpatients and prevalence of any coercive measures among inpatients by admission year and region. Table S4. Estimates of all parameters by multilevel logistic regression models with random effects (SE in brackets). (DOCX 40 kb) [file 12888_2019_2200_MOESM1_ESM.docx]

**Supplementary data**

**Table 1. Number of inpatients and prevalence of coercive treatment methods among inpatients by admission year: raw data**

| Admission year | All inpatients | Female, % | Any coercive methods  Patients (%) | Seclusion  Patients (%) | Limb restraints  Patients (%) | Forced injection  Patients (%) | Physical restraints  Patients (%) |
| --- | --- | --- | --- | --- | --- | --- | --- |
| 1995 | 24979 | 45.5 | 2441 (9.77) | 1529 (6.12) | 873 (3.49) | 545 (2.18) | 260 (1.04) |
| 1996 | 25886 | 45.3 | 2571 (9.93) | 1625 (6.28) | 1023 (3.95) | 606 (2.34) | 267 (1.03) |
| 1997 | 26003 | 45.5 | 2367 (9.10) | 1533 (5.90) | 964 (3.71) | 576 (2.22) | 241 (0.93) |
| 1998 | 26000 | 45.9 | 2512 (9.66) | 1651 (6.35) | 1127 (4.33) | 679 (2.61) | 262 (1.01) |
| 1999 | 25901 | 46.7 | 2707 (10.50) | 1791 (6.91) | 1222 (4.72) | 754 (2.91) | 265 (1.02) |
| 2000 | 28503 | 47.3 | 2881 (10.10) | 1894 (6.64) | 1320 (4.63) | 779 (2.73) | 238 (0.84) |
| 2001 | 27951 | 47.6 | 2896 (10.04) | 2001 (7.16) | 1333 (4.77) | 794 (2.84) | 248 (0.89) |
| 2002 | 28235 | 46.8 | 2590 (9.17) | 1728 (6.12) | 1061 (3.76) | 745 (2.64) | 220 (0.78) |
| 2003 | 28028 | 48.9 | 2844 (10.20) | 1998 (7.13) | 1064 (4.09) | 743 (2.65) | 204 (0.73) |
| 2004 | 26586 | 48.2 | 2704 (10.20) | 1868 (7.03) | 1031 (3.88) | 702 (2.64) | 161 (0.61) |
| 2005 | 26200 | 48.9 | 2758 (10.50) | 2010 (7.67) | 1035 (3.95) | 675 (2.58) | 168 (0.64) |
| 2006 | 26084 | 50.1 | 2750 (10.50) | 2021 (7.75) | 1114 (4.27) | 640 (2.45) | 153 (0.59) |
| 2007 | 25615 | 49.3 | 2722 (10.60) | 2095 (8.18) | 924 (3.61) | 630 (2.46) | 127 (0.50) |
| 2008 | 26170 | 49.0 | 2575 (9.84) | 1961 (7.49) | 958 (3.66) | 597 (2.28) | 141 (0.54) |
| 2009 | 24689 | 48.7 | 2435 (9.87) | 1809 (7.33) | 820 (3.32) | 514 (2.08) | 107 (0.43) |
| 2010 | 23132 | 49.6 | 2219 (9.59) | 1668 (7.21) | 731 (3.16) | 492 (2.13) | 104 (0.45) |
| 2011 | 22630 | 49.5 | 2062 (9.11) | 1502 (6.64) | 650 (2.87) | 489 (2.16) | 140 (0.62) |
| 2012 | 22187 | 50.0 | 1989 (8.96) | 1426 (6.63) | 620 (2.79) | 572 (2.58) | 179 (0.62) |
| 2013 | 21096 | 50.8 | 1959 (9.29) | 1353 (6.41) | 645 (3.06) | 763 (3.62) | 337 (1.60) |
| 2014 | 19300 | 50.2 | 1745 (9.04) | 1257 (6.51) | 516 (2.67) | 597 (3.09) | 122 (0.63) |
| **Total** | **505169** | **48.1** | **49731 (9.84)** | **34720 (6.87)** | **19113 (3.78)** | **12892 (2.55)** | **3944 (0.78)** |

**Table 2 Prevalence of coercive treatment methods among inpatients by gender by admission year: raw data**

| Admission year | Male inpatients | | | | | |  | Female inpatients | | | | | |
| --- | --- | --- | --- | --- | --- | --- | --- | --- | --- | --- | --- | --- | --- |
|  | Any coercive % | | Sec- lusion % | Limb restraint % | Forced inject-ion % | Physical restraint % |  | Any coercive % | | Sec-lusion  % | Limb restraint % | Forced injection % | Physical restraint % |
| 1995 | 10.90 | 6.82 | | 4.31 | 2.05 | 1.1 |  | 8.42 | 5.29 | | 2.52 | 2.34 | 0.97 |
| 1996 | 10.81 | 6.95 | | 4.85 | 1.95 | 1.03 |  | 8.87 | 5.46 | | 2.87 | 2.82 | 1.03 |
| 1997 | 9.87 | 6.58 | | 4.52 | 1.95 | 0.90 |  | 8.18 | 5.08 | | 2.74 | 2.54 | 0.95 |
| 1998 | 10.50 | 6.95 | | 5.27 | 2.47 | 1.01 |  | 8.72 | 5.58 | | 3.23 | 2.78 | 1.01 |
| 1999 | 11.40 | 7.62 | | 5.89 | 2.67 | 1.10 |  | 9.42 | 6.11 | | 3.38 | 3.19 | 0.93 |
| 2000 | 10.90 | 7.38 | | 5.53 | 2.34 | 0.84 |  | 9.21 | 5.83 | | 3.63 | 3.17 | 0.82 |
| 2001 | 11.40 | 7.93 | | 5.61 | 2.67 | 0.89 |  | 9.26 | 6.30 | | 3.85 | 3.03 | 0.89 |
| 2002 | 9.85 | 6.87 | | 4.44 | 2.31 | 0.72 |  | 8.40 | 5.27 | | 2.98 | 3.01 | 0.85 |
| 2003 | 11.10 | 8.31 | | 5.04 | 2.42 | 0.73 |  | 8.83 | 5.89 | | 3.09 | 2.89 | 0.73 |
| 2004 | 11.99 | 8.61 | | 4.86 | 2.6 | 0.54 |  | 8.22 | 5.33 | | 2.82 | 2.68 | 0.68 |
| 2005 | 12.70 | 9.46 | | 5.31 | 2.48 | 0.60 |  | 8.25 | 5.80 | | 2.53 | 2.67 | 0.68 |
| 2006 | 12.47 | 9.36 | | 5.55 | 2.40 | 0.50 |  | 8.63 | 6.15 | | 3.00 | 2.51 | 0.67 |
| 2007 | 12.93 | 10.14 | | 4.73 | 2.59 | 0.53 |  | 8.26 | 6.16 | | 2.45 | 2.33 | 0.46 |
| 2008 | 11.25 | 8.69 | | 4.57 | 2.09 | 0.55 |  | 8.37 | 6.24 | | 2.72 | 2.48 | 0.52 |
| 2009 | 11.75 | 8.85 | | 4.30 | 1.88 | 0.36 |  | 7.88 | 5.72 | | 2.29 | 2.30 | 0.51 |
| 2010 | 11.42 | 8.68 | | 4.15 | 2.03 | 0.45 |  | 7.74 | 5.72 | | 2.15 | 2.22 | 0.44 |
| 2011 | 10.93 | 7.91 | | 3.78 | 2.23 | 0.67 |  | 7.26 | 5.34 | | 1.95 | 2.09 | 0.56 |
| 2012 | 10.73 | 8.08 | | 3.64 | 2.63 | 0.73 |  | 7.20 | 4.77 | | 1.95 | 2.52 | 0.88 |
| 2013 | 11.00 | 7.76 | | 4.04 | 3.69 | 1.77 |  | 7.66 | 5.12 | | 2.11 | 3.54 | 1.43 |
| 2014 | 10.60 | 8.10 | | 3.42 | 3.16 | 0.47 |  | 7.50 | 4.94 | | 1.93 | 3.03 | 0.80 |
| **Total** | **11.23** | **8.02** | | **4.75** | **2.41** | **0.78** |  | **8.35** | **5.63** | | **2.74** | **2.71** | **0.79** |

**Table 3. Number of inpatients and prevalence of any coercive treatment methods among inpatients by admission year and region**

| Admission year | Southern Finland  Patients (%) | Western Finland  Patients (%) | Eastern Finland  Patients (%) | Northern Finland  Patients (%) | Åland  Patients (%) |
| --- | --- | --- | --- | --- | --- |
| 1995 | \| 987 (8.03) \| \| --- \| | \| 611 (11.18) \| \| --- \| | \| 558 (14.11) \| \| --- \| | \| 285 (8.99) \| \| --- \| | \| 0 (0.00) \| \| --- \| |
| 1996 | \| 1073 (8.32) \| \| --- \| | \| 640 (11.49) \| \| --- \| | \| 575 (14.40) \| \| --- \| | \| 275 (8.29) \| \| --- \| | \| 8 (7.62) \| \| --- \| |
| 1997 | \| 1008 (7.74) \| \| --- \| | \| 560 (10.00) \| \| --- \| | \| 514 (12.84) \| \| --- \| | \| 280 (8.52) \| \| --- \| | \| 5 (5.68) \| \| --- \| |
| 1998 | \| 1179 (8.96) \| \| --- \| | \| 563 (10.15) \| \| --- \| | \| 488 (12.53) \| \| --- \| | \| 279 (8.41) \| \| --- \| | \| 3 (3.41) \| \| --- \| |
| 1999 | \| 1382 (10.54) \| \| --- \| | \| 581 (10.33) \| \| --- \| | \| 472 (12.21) \| \| --- \| | \| 271 (8.52) \| \| --- \| | \| 1 (0.83) \| \| --- \| |
| 2000 | \| 1595 (10.29) \| \| --- \| | \| 549 (9.98) \| \| --- \| | \| 466 (11.01) \| \| --- \| | \| 271 (8.31) \| \| --- \| | \| 0 (0.00) \| \| --- \| |
| 2001 | \| 1577 (10.55) \| \| --- \| | \| 584 (10.45) \| \| --- \| | \| 455 (11.68) \| \| --- \| | \| 279 (7.98) \| \| --- \| | \| 1 (4.00) \| \| --- \| |
| 2002 | \| 1461 (9.90) \| \| --- \| | \| 472 (8.10) \| \| --- \| | \| 396 (9.94) \| \| --- \| | \| 257 (7.32) \| \| --- \| | \| 4 (2.48) \| \| --- \| |
| 2003 | \| 1666 (11.22) \| \| --- \| | \| 534 (9.04) \| \| --- \| | \| 450 (11.67) \| \| --- \| | \| 195 (5.96) \| \| --- \| | \| 3 (2.04) \| \| --- \| |
| 2004 | \| 1375 (10.80) \| \| --- \| | \| 597 (9.53) \| \| --- \| | \| 495 (12.16) \| \| --- \| | \| 229 (6.77) \| \| --- \| | \| 8 (5.71) \| \| --- \| |
| 2005 | \| 1388 (11.32) \| \| --- \| | \| 651 (10.36) \| \| --- \| | \| 487 (11.85) \| \| --- \| | \| 222 (6.56) \| \| --- \| | \| 10 (6.21) \| \| --- \| |
| 2006 | \| 1393 (11.35) \| \| --- \| | \| 608 (9.88) \| \| --- \| | \| 478 (11.79) \| \| --- \| | \| 265 (7.69) \| \| --- \| | \| 6 (4.00) \| \| --- \| |
| 2007 | \| 1379 (11.46) \| \| --- \| | \| 647 (10.52) \| \| --- \| | \| 466 (12.14) \| \| --- \| | \| 225 (6.53) \| \| --- \| | \| 5 (3.42) \| \| --- \| |
| 2008 | \| 1243 (10.08) \| \| --- \| | \| 641 (10.19) \| \| --- \| | \| 453 (11.66) \| \| --- \| | \| 231 (6.58) \| \| --- \| | \| 7 (4.58) \| \| --- \| |
| 2009 | \| 1275 (10.68) \| \| --- \| | \| 604 (10.38) \| \| --- \| | \| 339 (9.44) \| \| --- \| | \| 199 (6.22) \| \| --- \| | \| 18 (13.14) \| \| --- \| |
| 2010 | \| 1185 (10.55) \| \| --- \| | \| 547 (9.87) \| \| --- \| | \| 298 (9.37) \| \| --- \| | \| 178 (5.84) \| \| --- \| | \| 11 (8.27) \| \| --- \| |
| 2011 | \| 1100 (10.13) \| \| --- \| | \| 478 (8.56) \| \| --- \| | \| 285 (9.67) \| \| --- \| | \| 187 (6.04) \| \| --- \| | \| 12 (8.28) \| \| --- \| |
| 2012 | \| 975 (9.31) \| \| --- \| | \| 520 (9.45) \| \| --- \| | \| 260 (9.01) \| \| --- \| | \| 222 (7.03) \| \| --- \| | \| 12 (7.14) \| \| --- \| |
| 2013 | \| 925 (9.23) \| \| --- \| | \| 507 (9.87) \| \| --- \| | \| 275 (9.56) \| \| --- \| | \| 241 (8.22) \| \| --- \| | \| 11 (8.66) \| \| --- \| |
| 2014 | \| 871 (9.29) \| \| --- \| | \| 420 (9.14) \| \| --- \| | \| 242 (9.18) \| \| --- \| | \| 201 (7.89) \| \| --- \| | \| 11 (7.80) \| \| --- \| |

**______________________________________________________________________________________**

**Table 4 Estimates of all parameters by multilevel logistic regression models with random effects (SE in brackets)**

|  | **Any coercive method** | **Seclusion** | **Limb restraints** | **Forced injection** | **Physical restraints** |
| --- | --- | --- | --- | --- | --- |
| **Fixed Part** |  |  |  |  |  |
| Intercept | **-5.43(.795)‡** | **-5.92(.907) ‡** | **-6.49(1.04)‡** | **-7.62(.919)‡** | **-5.55(.757)‡** |
| Year | -.0014(.0136) | .012(.014) | -.014(.020) | .016(.016) | **-.043(.022)*** |
| Year^2 | **-.0057(.0019)†** | **-.0072(.0020)†** | **-.014(.0006)‡** | **-.0057(.0023)*** | **-.0089(.0026)†** |
| Region_2 | -.500(.323) | -.588(.367) | -.693(.400) | -.458(.366) | **-.149(.318)†** |
| Region_3 | .276(.339) | .263(.384) | .915(.415) | .071(.378) | .363(.337) |
| Region_4 | **-.898(.374)** | **-1.85(.433)** | -.436(.462) | **-1.46(.428)†** | -.639(.374) |
| Region_5 | -.293(.920) | -.306(1.07) | -1.03(1.20) | .255(1.07) | .317(.996) |
| Year.Region_2 | .0006(.026) | .0021(.026) | -.015(.040) | -.0062(.028) | .039(.037) |
| Year^2.Region_2 | .0045(.0037) | .0065(.0040) | **.010(.0010)*** | .0077(.0043) | **.018(.0044)†** |
| Year.Region_3 | .0031(.028) | -.0066(.029) | -.0086(.039) | -.025(.031) | -.039(.041) |
| Year^2.Region_3 | .0025(.0038) | .000032(.0041) | **.014(.0009)‡** | .0054(.0045) | .0031(.0048) |
| Year.Region_4 | .0085(.030) | .0309(.032) | .029(.044) | -.044(.034) | -.032(.042) |
| Year^2.Region_4 | .0068(.0041) | **.0092(.0046)*** | **.015(.001)‡** | **.010(.0045)*** | **.014(.0051)*** |
| Year.Region_5 | .079(.078) | .081(.080) | .220(.131) | .111(.084) | -.111(.097) |
| Year^2.Region_5 | .011(.011) | .011(.011) | .015(.012) | .012(.012) | .017(.012) |
| Female | **-.357(.014)‡** | **-.396(.017)‡** | **-.608(.022)‡** | **.093(.027)‡** | .012(.050) |
| Year.Female | **-.013(.002)‡** | **-.016(.002)‡** | **-.013(.003)‡** | **-.017(.003)‡** | .0063(.0055) |
| Year^2.Female | **.0008(.0003)†** | **.0009(.0004)†** | .0005(.0006) | -.0007(.0062) | -.0008(.0011) |
| Age | **-.0028(.0003)‡** | **-.0079(.0004)‡** | **-.0113(.0005)‡** | **.0036(.0006)‡** | **.0083(.0010)‡** |
| Treatment periods | **.380(.094) ‡** | **.394(.107)†** | **.414(.122)†** | **.433(.108)†** | .038(.088) |
| **Random Part** |  |  |  |  |  |
| **Level: Care provider** |  |  |  |  |  |
| Intercept/Intercept | 1.14(.186)**‡** | 1.45(.238)**‡** | 1.68(.281)**‡** | 1.31(.227) **‡** | 0.931(.176)**‡** |
| Year/Intercept | -.018(.011) | -.032(.012) | .0087(.019) | -.0071(.013) | -.014(.015) |
| Year/Year | .0057(.0011)**‡** | .0056(.0012)***** | .129(.025)**‡** | .0058(.0013)**†** | .010(.002)**‡** |
| Year^2/Intercept | .0018(.0015) | .024(.0018) |  | -.0055(.0020)***** | .0063(.0019)**†** |
| Year^2/Year | -.0006(.0001)**‡** | -.0004(.0001)***** |  | -.0004(.00015)**†** | .00017(.00019) |
| Year^2/Year^2 | .00012(.00002)**‡** | .00013(.00003)**‡** |  | .00013(.00003)**‡** | .00011(.000031)**†** |

**‡p<0.001, †p<0.01, *p<0.05.**
